# RETRACTED: Mijiritsky et al. Porcine Bone Scaffolds Adsorb Growth Factors Secreted by MSCs and Improve Bone Tissue Repair. *Materials* 2017, *10*, 1054

**DOI:** 10.3390/ma19143124

**Published:** 2026-07-21

**Authors:** Eitan Mijiritsky, Letizia Ferroni, Chiara Gardin, Eriberto Bressan, Gastone Zanette, Adriano Piattelli, Barbara Zavan

**Affiliations:** 1 Department of Otolaryngology, Head and Neck and Maxillofacial Surgery, Sackler Faculty of Medicine, Tel-Aviv Sourasky Medical Center, Tel Aviv University, 6 Weitzman Street, Tel Aviv 64239, Israel; 2 Department of Biomedical Sciences, University of Padova, via G. Colombo 3, 35100 Padova, Italy; 3 Department of Neurosciences, University of Padova, via Giustiniani 5, 35100 Padova, Italy; 4 Department of Medical, Oral, and Biotechnological Sciences, University of Chieti-Pescara, via dei Vestini 31, 66100 Chieti, Italy; 5 Maria Cecilia Hospital, GVM Care & Research, Cotignola, 48033 Ravenna, Italy

The journal retracts the article titled “Porcine Bone Scaffolds Adsorb Growth Factors Secreted by MSCs and Improve Bone Tissue Repair” [1], cited above.

Following publication, concerns were brought to the attention of the Editorial Office regarding the integrity of some images presented in this publication [1].

In accordance with standard journal procedures, the Editorial Office and Editorial Board conducted an investigation that confirmed a duplication between Figure 6C in [1] and Figure 5C in [2], presenting different experimental conditions. While the authors collaborated during the investigation, they were unable to satisfactorily address the concerns.

As a result, the Editorial Board has lost confidence in the reliability of the findings and has decided to retract this publication, as per MDPI’s retraction policy (https://www.mdpi.com/ethics#_bookmark30).

This retraction was approved by the Editor-in-Chief of the journal *Materials*.

The authors have been informed of this retraction and have indicated their disagreement with this decision.
